# Health-related quality of life and clinical outcome after radiotherapy of patients with intracranial meningioma

**DOI:** 10.1038/s41598-022-24192-8

**Published:** 2022-11-17

**Authors:** Dominik Lisowski, Jannik Trömel, Paul Lutyj, Victor Lewitzki, Philipp E. Hartrampf, Bülent Polat, Michael Flentje, Jörg Tamihardja

**Affiliations:** 1 Department of Radiation Oncology, University Hospital Wuerzburg, Josef-Schneider-Str. 11, 97080 Wuerzburg, Germany; 2 Department of Internal Medicine, Leopoldina Hospital Schweinfurt, Schweinfurt, Germany; 3 Department of Nuclear Medicine, University Hospital Wuerzburg, Wuerzburg, Germany

**Keywords:** Outcomes research, CNS cancer, Radiotherapy

## Abstract

This retrospective, single-institutional study investigated long-term outcome, toxicity and health-related quality of life (HRQoL) in meningioma patients after radiotherapy. We analyzed the data of 119 patients who received radiotherapy at our department from 1997 to 2014 for intracranial WHO grade I-III meningioma. Fractionated stereotactic radiotherapy (FSRT), intensity modulated radiotherapy (IMRT) or radiosurgery radiation was applied. The EORTC QLQ-C30 and QLQ-BN20 questionnaires were completed for assessment of HRQoL. Overall survival (OS) for the entire study group was 89.6% at 5 years and 75.9% at 10 years. Local control (LC) at 5 and 10 years was 82.4% and 73.4%, respectively. Local recurrence was observed in 22 patients (18.5%). Higher grade acute and chronic toxicities were observed in seven patients (5.9%) and five patients (4.2%), respectively. Global health status was rated with a mean of 59.9 points (SD 22.3) on QLQ-C30. In conclusion, radiotherapy resulted in very good long-term survival and tumor control rates with low rates of severe toxicities but with a deterioration of long-term HRQoL.

Meningiomas are neoplasms derived from the arachnoidal cells of the leptomeninges and are the most common primary intracranial tumors in adults with 15–30%^1^. Women are twice as often affected as men, however, men tend to develop more aggressive forms of meningiomas^2^. Meningiomas are classified in three groups by the World Health Organization (WHO) according to histological characteristics. Approximately 80–85% of all meningiomas are categorized as non-malignant meningiomas (WHO grade I), which commonly exhibit a slow growth rate and a noninvasive expansion. Only 5–15% of meningiomas are considered as atypical meningiomas (WHO grade II) and only 1–3% are malignant meningiomas (WHO grade III) with a tendency of brain invasion^3,4^. A novel meningioma classification is based on molecular markers to predicted clinical outcomes more accurately^5^. Multimodal meningioma treatment is dependent on WHO grading as well as resection status and may include surgery, radiotherapy, peptide receptor radionuclide therapy (PRRT) or watchful waiting^6^. Radiotherapy is commonly applied as adjuvant therapy or in relapse situation. In case of unresectable meningioma, primary radiotherapy is the most common treatment option. It may be conducted in terms of intensity modulated radiotherapy (IMRT), fractionated stereotactic radiotherapy (FSRT) or radiosurgery^7^. Although modern radiation techniques have decreased the amount and severity of acute and late toxicity, previous publications observed high-grade adverse effects after radiotherapy of the brain such as visual field deficit, neuropathy, cerebral necrosis, pituitary dysfunction and cerebrovascular events^8–13^. Cognitive impairment, memory loss and personality changes might be objectively difficult to quantify, but have a huge impact on daily life of individuals^9,14^. Consequently, any acute or late side effect may lead to a significant deterioration in health-related quality of life (HRQoL)^15^. Up to date, few data has been published for toxicity and HRQoL after radiotherapy in meningioma patients. Therefore, the present retrospective single-center analysis aimed to provide data on long-term HRQoL, side effects and efficacy after radiotherapy in a large group of meningioma patients.

## Methods

We retrospectively analyzed data from 119 consecutive meningioma patients who were treated at our department between 1997 and 2014. Ethical approval was waived by the local Ethics Committee of the University of Wuerzburg in view of the retrospective nature of the study and all the procedures being performed were part of the routine care. All methods were carried out in accordance with the ethical standards of the institutional research committee and the 1964 Helsinki Declaration and its later amendments or comparable ethical standards. Informed consent was obtained from all subjects and/or their legal guardian(s) before treatment planning. The primary endpoint of our study was HRQoL, assessed by the European Organisation for Research and Treatment of Cancer (EORTC) Quality of Life Core questionnaire (QLQ-C30) version 3.0 and the EORTC Brain Cancer Module questionnaire (QLQ-BN20). Secondary endpoints were treatment related toxicity, 5- and 10-year local control (LC) and overall survival (OS). LC was defined as the time between initiation of radiation and the occurrence of first progression at the treated site on imaging. OS was defined as the time between diagnosis and the last documented follow-up or death from any cause. Included were all patients with meningioma who were treated with radiotherapy in the given time period and who showed no signs of spinal infiltration. In case of multiple treatment series, we analyzed one series only. Patients with history of a different cancer, independently of previous treatment, were included in the database. All cases were discussed in an interdisciplinary neuro-oncological review board before treatment.

### Treatment planning

For normofractionated radiotherapy, the gross tumor volume (GTV) was expanded by 8–15 mm, depending on the WHO grade and tumor location, to generate the clinical target volume (CTV). The CTV was expanded by 3 mm resulting in the planning target volume (PTV). Dose was prescribed to the mean PTV dose. In case of stereotactic radiotherapy, a margin of 1–2 mm was added to the GTV for the PTV. IMRT was delivered as a step-and-shoot technique with 3–9 fields or as Volumetric Intensity Modulated Arc Therapy (VMAT) with two dynamic arcs. All radiation therapies were conducted with photon beams using an ELEKTA Synergy® or a Siemens PRIMUS linear accelerator. GTV was contoured on a computed tomography (CT) scan with co-registered MR imaging using Pinnacle^3^ (Philips Radiation Oncology Systems, Fitchburg, WI, USA).

### Follow-up

Clinical and radiologic follow-up including contrast-enhanced MRI was performed 6–12 weeks after radiation therapy and thereafter once or twice per year, unless an earlier examination was considered due to suspected relapse. Imaging examinations were assessed by two independent (neuro)radiologists. Tumor dimensions were measured according to an axial T1-weighted contrast-enhanced MRI sequence or to a contrast enhanced CT scan. In case of multifocal occurrence, tumor location was defined by the site of the largest lesion. Response Assessment in Neuro-Oncology (RANO) criteria were used to evaluate tumor progression^16^. Tumor localization was categorized in skull base, cerebral falx, hemispheral convexity or optic nerve sheath.

Clinical examination included assessment of neurological status. For the evaluation of acute and late toxicity, the Common Terminology Criteria for Adverse Events (CTCAE) version 5.0 was used. Acute toxicity was assessed up to 90 days after the end of radiation. For HRQoL assessment, EORTC QLQ-C30 version 3.0 and the EORTC QLQ-BN20 were filled out at follow-up visits or were sent out to the patients. Karnofsky Performance Status (KPS) was assessed before treatment and at time of HRQoL assessment. QLQ-C30 and QLQ-BN20 data was compared with already published data of historic cohorts^17–23^.

### Statistics

All data were analyzed with IBM SPSS Statistics 26.0. The threshold for statistical significance was set at a two-sided *p* < 0.05. We assumed that data in our performed study is missing at random (MAR). As only very few values were missing, we used pairwise deletion to sustain a sufficiently large sample size and power. Regarding the QLQ-C30 and BN20 questionnaires, a relevant clinical difference was defined when the point difference was greater than 10 points^24,25^. OS and LC were calculated using Kaplan–Meier statistics. Log-rank testing was used to determine the statistical significance of the OS or LC difference between different groups. For multivariate analysis, Cox proportional hazard regression was performed. Mann–Whitney-U and Kruskal–Wallis tests were performed due to not normally distributed parameters according to the Shapiro–Wilk test. To correlate toxicity grades with treatment characteristics, tumor location and HRQoL data, the significance of Kendall’s tau-b correlation coefficient was assessed. Multiple linear regressions were used to find confounders for global health status.

## Results

### Treatment results

Meningioma was histologically determined in 76 patients (63.9%). In 43 patients (36.1%) diagnosis was based on radiologic signs after magnetic resonance imaging (MRI) examination by at least two (neuro)radiologists. Radiation was administered in 56 patients (47.0%) at initial diagnosis with 41 patients (34.5%) being irradiated within a year of diagnosis. The other 63 patients (52.9%) were treated at time of meningioma relapse. In total, 37 patients received a primary radiotherapy due to unresectability of the meningioma or inoperability which was conditioned by age and comorbidities. The median age of patients receiving primary, adjuvant and recurrent radiotherapy was 70.2, 58.4 and 56.7 years, respectively. Median tumor axial size was 2.5 cm (IQR 1.5–3.8 cm) in the longest orientation at the start of radiotherapy. FSRT, IMRT and radiosurgery alone were performed in 67 (56.3%), 48 (40.3%) and four (3.3%) patients, respectively. Sequential boost radiation was administered in 38 patients (31.9%). A median total dose of 54.0 Gy (IQR 54.0–58.5 Gy), 60.0 Gy (IQR 54–61.2 Gy) and 60.0 Gy (IQR 59.4–60.3 Gy) was administered for WHO grade I, II and III meningiomas, respectively. For stereotactic radiotherapy, a median total dose of 19.5 Gy (range 17.5–21 Gy) was prescribed to the 68% PTV encompassing isodose. One patient was treated with whole brain irradiation with 30.0 Gy and a sequential boost on the meningioma lesion with 15.0 Gy. In 26 patients (21.8%), (68)Ga-DOTA^0^-Phe^1^-Tyr^3^ octreotide (DOTATOC), (68)Ga-DOTA^0^-Tyr^3^ octreotate (DOTATATE) or (18)F-Fluoroethyl-L-tyrosine (FET) positron emission tomography (PET) imaging was performed and fused for improved target volume definition. Nine patients (7.6%) received an additional PRRT using (177)Lu-DOTATOC with a mean dose of 7.5 Gy (SD ± 0.3). Three patients received a concomitant or sequential chemotherapy. All patients’ characteristics are summarized in Table 1.

**Table 1:** Patient characteristics (n = 119).

Variable	Number	(%)
**Gender**		
Male	43	36.1%
Female	76	63.9%
**Age**		
Mean (SD)	58.7	(14.2)
**Localization**		
Skull base	73	61.3%
Cerebral falx	27	22.7%
Hemispheral convexity	15	12.6%
Optic nerve sheath	4	3.4%
**Histology**		
No specimen collected	43	36.1%
WHO Grade I	38	31.9%
WHO Grade II	20	16.8%
WHO Grade III	18	15.1%
**Karnofsky performance status**		
Median (range)	80	(30–100)
KPS ≥ 90%	59	49.6%
KPS < 90%	60	50.4%
**Simpson resection grade (n = 79)**		
Not known	7	5.9%
Grade I	6	5.0%
Grade II	12	10.1%
Grade III	2	1.7%
Grade IV	49	41.2%
Grade V	3	2.5%
**Treatment**		
Primary radiation	37	31.1%
Adjuvant radiation	19	16.0%
Relapse radiation	63	52.9%
**Radiation modalities**		
FSRT	67	56.3%
IMRT	48	40.3%
SRS	3	2.5%
WBRT	1	0.8%
**Radiopeptide therapy**		
Yes	9	7.6%
No	110	92.4%

FSRT = fractionated stereotactic radiotherapy; IMRT = intensity modulated radiotherapy; SD = standard deviation; SRS = stereotactic radiosurgery; WBRT = whole brain radiotherapy; WHO = World Health Organization.

### Quality of life

**Figure 1:**
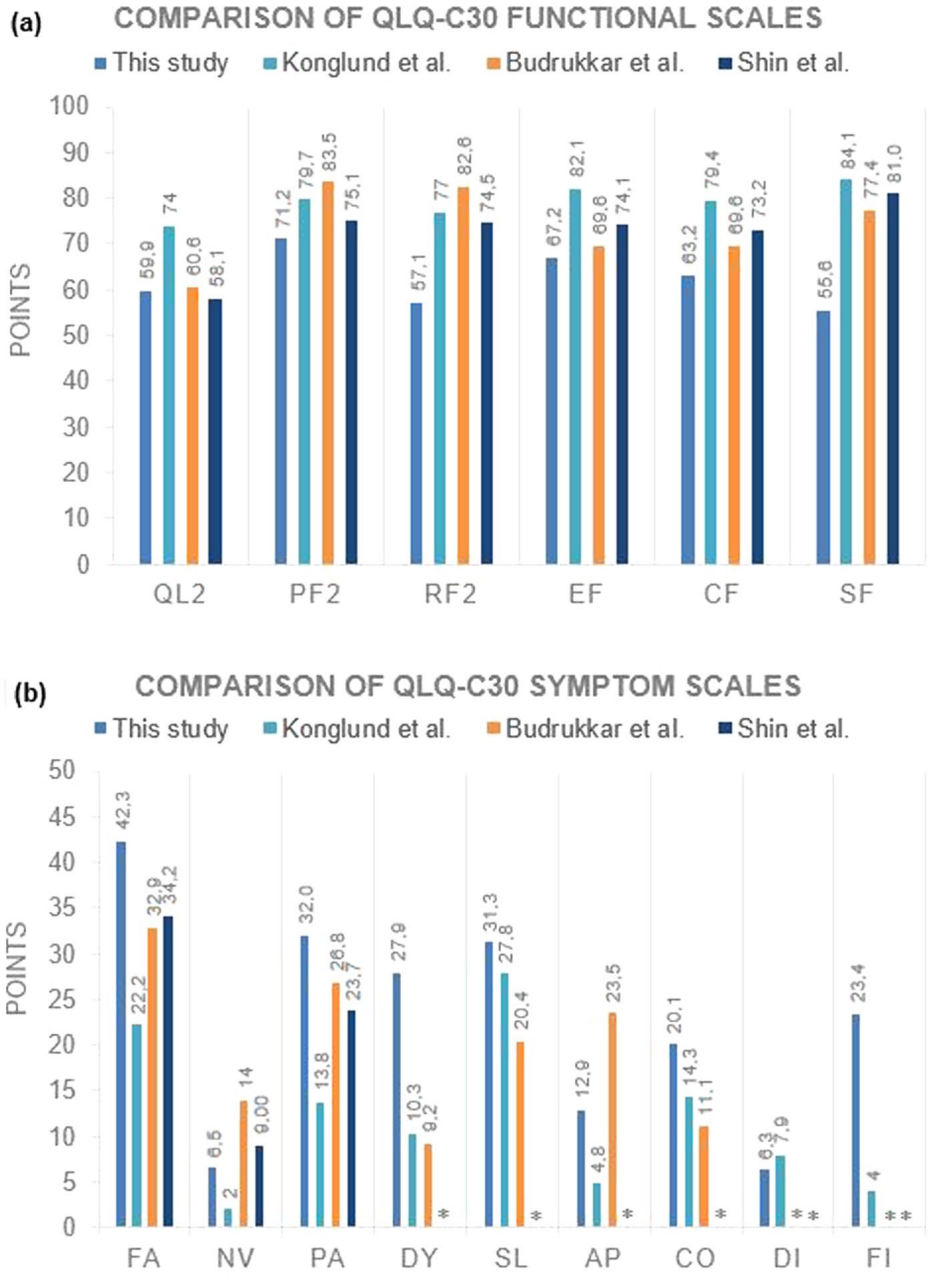
(**a**) Comparison of QLQ-C30 functional scales with previously published data. Higher scores in functional domains suggest higher level of functioning and better quality of life. QL2 = Global health status (revised); PF2 = Physical functioning (revised); RF2 = Role functioning (revised); EF = Emotional functioning; CF = Cognitive functioning; SF = Social functioning. (**b**) Comparison of QLQ-C30 symptom scales with previously published data. Higher scores in symptomatic domains suggest lower level of functioning and worse quality of life. *Abbreviations:* FA = Fatigue; NV = Nausea and vomiting; PA = Pain; DY = Dyspnea; SL = Insomnia; AP = Appetite loss; CO = Constipation; DI = Diarrhea; FI = Financial difficulties. *Data not published.

Since 37 patients (31.1%) have already died at time of survey, 82 questionnaires were forwarded or sent out, from which 49 were appropriately filled out and returned, resulting in a response rate of 59.8%. One questionnaire was returned with the notification that the corresponding patient had died. The median KPS of the surveyed patients before radiotherapy and at time of HRQoL assessment was 90 (range 50–100) and 90 (range 40–100), respectively. The median KPS of the entire study group was 80 (range 30–100). The questionnaires were completed in median 4.8 years (IQR 2.7–9.2 years) after radiotherapy from patients with a median age of 64.4 years (IQR 59.0–72.5 years). Out of the 49 responders, three received additional cranial and four extracranial radiotherapy sequentially. Baseline patient characteristics analysis between the responding and non-responding groups was not significant except for PRRT (Supplementary Table S1). In terms of self-assessment, the global health status was rated with a mean of 59.9 points (SD 22.3) on the EORTC QLQ-C30 with functional scales ranging between a mean of 55.6 and 71.2 points (Table 2). We could detect a relevant decrease on the functional scale for physical, role, cognitive and social functioning, which was accompanied by an increase on the symptom scale for fatigue, pain, dyspnea, insomnia, constipation and financial impact. There was no statistically significant correlation between maximal chronic toxicity grade and fatigue (*p* = 0.41), nausea and vomiting (*p* = 0.43), pain (*p* = 0.12), dyspnea (*p* = 0.5), insomnia (*p* = 0.35), appetite loss (*p* = 0.20), constipation (*p* = 0.09), diarrhea (*p* = 0.49) nor financial difficulties (*p* = 0.46). Sequential radiotherapies, metachronous secondary malignancies and localization of the meningioma were not confounders for global health status on the QLQ-C30 (*p* ≥ 0.05). On the EORTC QLQ-BN20, the most common impairments were drowsiness, uncertainty about the future and weakness of the legs (Table 2). Compared to previous cohorts, our data showed partially worse results on the QLQ-C30 (Fig. 1) and on the QLQ-BN20 (Supplementary Fig. S1).

**Table 2:** Scores of EORTC QLQ-C30 and BN20 items (n = 49).

	Scale	Mean	SD
**QLQ-C30**			
Global health status	QL2	59.9	22.3
**Functional scales**			
Physical functioning	PF2	71.2	28.8
Role functioning	RF2	57.1	35.0
Emotional functioning	EF	67.2	27.4
Cognitive functionin	CF	63.2	33.0
Social functioning	SF	55.6	38.3
**Symptom scales**			
Fatigue	FA	42.3	31.8
Nausea and vomiting	NV	6.5	16.6
Pain	PA	32.0	34.5
Dyspnea	DY	27.9	33.6
Insomnia	SL	31.3	32.2
Appetite loss	AP	12.9	25.3
Constipation	CO	20.1	31.9
Diarrhea	DI	6.3	16.2
Financial difficulties	FI	23.4	32.9
**QLQ-BN20**			
Future uncertainty	BNFU	37.9	30.5
Visual disorder	BNVD	21.3	25.5
Motor dysfunction	BNMD	29.5	30.1
Communication deficit	BNCD	22.2	25.8
Headaches	BNHA	29.3	30.9
Seizures	BNSE	6.1	20.0
Drowsiness	BNDR	42.2	34.5
Itchy skin	BNIS	19.0	29.7
Hair loss	BNHL	19.0	31.2
Weakness of legs	BNWL	30.6	36.5
Bladder control	BNBC	20.8	29.5

EORTC = European Organisation for Research and Treatment of Cancer; QLQ-BN20 = Quality of Life Questionnaire brain cancer module; QLQ-C30 = Core Quality of Life Questionnaire; SD = standard deviation.

### Side effects

Radiation-related acute toxicities with clinical significance (CTCAE grade ≥ 3) were recorded in seven patients (5.9%). These included one case of amaurosis with prior visibility impairment and three cases of dizziness. Other CTCAE grade 3 toxicities were nausea, headache, radiation dermatitis, fatigue and mucositis. In two cases, the irradiation had to be discontinued due to a deterioration of general health. Acute grade 1 and 2 side effects occurred in 52.9% and 37.8% of cases, respectively. Fatigue, alopecia, headache, radiation dermatitis, dizziness, as well as nausea and vomiting were the most common acute side effects reported. In 3.4% of all cases, no adverse effects were reported.

Severe chronic toxicities (CTCAE grade ≥ 3) were observed in five patients (4.2%). There was one case of surditas (CTCAE grade 4) and one case of amaurosis (CTCAE grade 4) with anterior pituitary insufficiency (CTCAE grade 3). The other three cases suffered from tiredness, exhaustion, confusion or headache (all CTCAE grade 3). Chronic side effects of CTCAE grade 1 and 2 were found in 11.8% and 20.2% of the patients, respectively. The most common chronic side effect was chronic headache, which occurred in 7.5% of all cases. In addition, circumscribed CNS toxicity (6.7%), memory and concentration disorders (5.9%) as well as fatigue (5.8%) were relatively common.

All mean dose values for organs at risk in our study group were below recommended limits. Patients with toxicities grade ≤ 2 received a mean total dose of 54.9 Gy. The mean total dose in patients with toxicities grade ≥ 3 was 57.9 Gy. There was no statistically significant correlation between maximal toxicity grade and total dose (p = 0.55), PTV (p = 0.86), GTV (p = 0.52) nor tumor location (p = 0.56). There was a statistically significant correlation between the acute fatigue toxicity grade and the fatigue symptom scale of QLQ-C30 (p = 0.03).

### Local control

**Figure 2:**
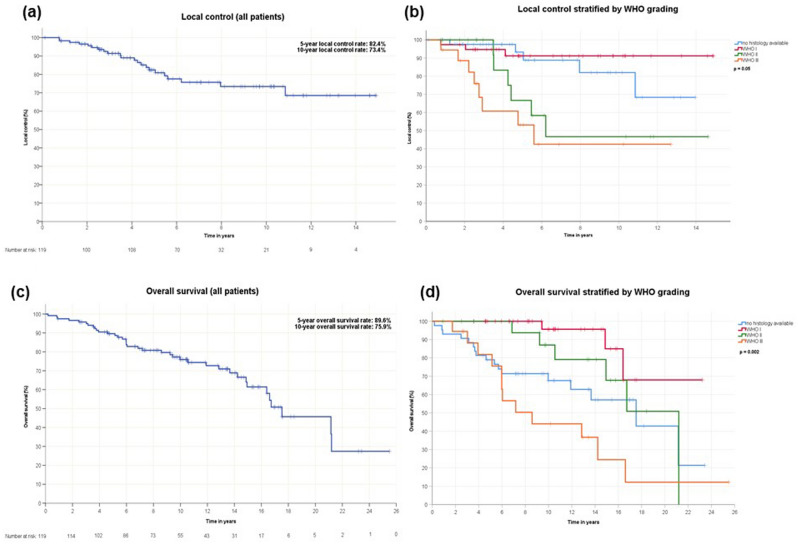
Local control shown by Kaplan–Meier analysis for all patients (**a**) and stratified by the WHO grading (**b**). WHO grading was suggestive for influencing local control (*p* = 0.05). Overall survival shown by Kaplan–Meier analysis for all patients (**c**) and stratified by the WHO grading (**d**). WHO grading was highly significant for overall survival (*p* = 0.002).

Median follow-up was 5.4 years (IQR 2.9–9.7 years). Estimated 5- and 10-year LC rates were 82.4% and 73.4%, respectively (Fig. 2a). The median time to recurrence was not reached at time of data analysis. In total, 22 patients (18.5%) had an in-field relapse, three patients with WHO grade I, six with WHO grade II and eight with WHO grade III meningiomas. A relapse also occurred in five patients without histologically confirmed meningioma. One patient with highly suspected neurofibromatosis type II was diagnosed with a meningioma relapse twice. The histological grade was significant and suggestive for influencing LC in the univariate (*p* < 0.001) and multivariate analysis (*p* = 0.05), respectively (Fig. 2b). Simpson grade (I-III vs. IV-V) did not have a statistically significant impact on LC. Location of the tumor (*p* = 0.032) as well as GTV for the subgroup of patients with WHO grade II and III meningiomas (*p* = 0.023) were significant in univariate analysis, but not in multivariate analysis. No significant difference in LC could be observed when comparing a cumulative dose of ≥ 60 Gy versus < 60 Gy for all patients (*p* = 0.37) nor for patients with WHO grade II and III meningiomas (*p* = 0.46).

### Overall survival

In total, 38 patients have died at time of survey. Out of the 38 deaths, 16 patients (42.1%) were presumed to have died from meningioma disease and ten patients (26.3%) succumbed to comorbidities. In 12 cases (31.6%), the cause of death remained unclear. Estimated 5- and 10-year OS was 89.6% and 75.9%, respectively (Fig. 2c). The median OS was 17.5 years. Survival rates significantly differed by WHO grade (*p* = 0.002). KPS (≥ 90% vs. < 90%) (*p* = 0.046), GTV (*p* = 0.001), timing of radiation (*p* = 0.005) and age (*p* = 0.001) had a significant impact on OS in univariate analysis. After multivariate analysis, WHO grade (*p* = 0.002) and GTV (*p* = 0.001) remained significant for OS (Fig. 2d). OS was not significantly affected by gender, Simpson grade, location of tumor and tumor volume before treatment. As for LC, no significant difference in OS was found comparing radiotherapies with a dose escalation above 60 Gy for all patients (*p* = 0.32) nor for patients with WHO grade II and III meningiomas (*p* = 0.08).

## Discussion

To the best of our knowledge, this study is the first one to assess HRQoL data using QLQ-C30 and BN20 questionnaires for exclusively meningioma patients who received radiotherapy. Our database search found only few publications evaluating HRQoL using QLQ-C30 and BN20 questionnaires in meningioma patients, mostly as unplanned subgroup analyses^17–19,23^. Response rate for HRQoL assessment was 59.8%. We could not detect a specific reason for the missing return of the 32 questionnaires and we can only speculate as to why the response rate was limited. Although we could not find a significant difference between the responding and non-responding groups except for PRRT (Supplementary Table S1), a selection bias cannot be excluded due to the limited response rate. Our data shows slightly lower HRQoL results in comparison to already published data for meningioma patients, although comparability might be limited due to different data acquisition methods and patient group compositions^26^. For instance, Erharter et al. performed a preselection of patients excluding patients with severe cognitive impairment, which resulted in higher HRQoL scores^23^. No additional information about meningioma patient group composition is provided by Shin et al.^19^. Budrukkar et al. assessed HRQoL in a subgroup of patients with benign brain tumors, which was not limited to meningioma patients only^18^. Konglund et al. reported higher QLQ-C30 scores which is probably attributable to group differences as their cohort consisted to 94% of benign, resected meningiomas without radiotherapy^17^. Primary radiotherapy is often chosen for advanced, inoperable tumors and benign meningiomas should not be irradiated after complete resection according to the European Association of Neuro-Oncology (EANO) guidelines^27,28^. The heterogeneity of our examined study group has to be taken into consideration. Our study group predominantly consisted of patients with unfavorable tumor locations and 100 patients received radiotherapy as first-line treatment or at time of relapse. Recurrent or incompletely resected meningiomas are prone to worse outcome with more clinically significant side effects and consequently lower HRQoL^13^. The rate of WHO grade II (16.8%) and III (15.1%) meningiomas in our study was higher than average, resulting in an overrepresentation of high-grade meningiomas (WHO grade II and III). In addition, 21 patients (17.6%) in our study group reported another malignant tumor before HRQoL assessment which might act as a confounder.

HRQoL was determined with a median time of 4.8 years after treatment in our study providing the possibility of other diseases negatively influencing HRQoL as confounders, such as stroke or cognitive deterioration due to aging. The lack of longitudinal assessment of HRQoL is a limiting factor of our analysis as HRQoL data was only assessed at a specific time point during follow-up. Hence, a pre-treatment survey is missing to compare HRQoL and to identify possible confounders or subgroups of patients with stronger HRQoL deterioration. Since only long-term HRQoL was assessed in our study, beneficial effects directly after radiotherapy or surgery resulting in functional gains and better HRQoL were not measured in contrast to the studies of Budrukkar et al., Konglund et al. and Bitterlich et al.^17,18,29^.

Physician-assessed severe acute toxicities appeared in only 5.9% of cases, confirming that radiotherapy has mild side effects when applied in meningioma patients. The one case of acute amaurosis could be attracted to tumor growth as the patient had severe visibility impairment prior to radiotherapy and received a palliative radiation with a lower dose. Albeit 36.2% of patients reported chronic toxicities, only 4.2% suffered from a chronic side effect CTCAE grade ≥ 3. Our findings are in line with previously published data in terms of acute and late toxicities (0–49.9%)^7,10,12,14,30,31^.

Our median applied dose of radiation was comparable with existing literature. Based on already published data, a dose of 54–60 Gy is indicated and well tolerated for WHO grade I meningiomas. In our WHO grade I meningioma cohort, a dose up to 66.0 Gy was accepted if histopathology specimens had angiomatous or fibrous components. For high-grade meningiomas, a median total dose of 60.0 Gy was prescribed in our study. A minimum dose of 60 Gy is usually prescribed for WHO grade III meningiomas to ensure long-term local control^32,33^. The dose prescription for WHO grade II meningiomas, however, is inconsistent throughout literature. Depending on the resection status, high dose radiation with 60 Gy or 70 Gy was prescribed for all WHO grade II meningioma patients in the EORTC 22042 study while newly diagnosed WHO grade II meningioma patients with gross total resection were treated with a lower radiation dose of 54 Gy in the RTOG 0539 study^34,35^. Three-year progression-free survival (PFS) and OS were comparable in both studies. Long-term results for both studies have not been published yet. In retrospective analyses, dose escalation, however, is associated with improved clinical outcome and may be prescribed for WHO grade II meningiomas^32,33,36,37^.

Existing reports on factors influencing OS and LC for meningioma are inconsistent except for WHO grade^11,15,30,38–41^. In line with these results, our data confirmed that the WHO grade had a significant impact on OS in univariate and multivariate analysis and affected local control as well. Due to the lack of studies with large patient numbers, statistics for OS and LC in WHO grade II and III meningiomas show a broad variance (0.0–89.0%) (Supplementary Table S2)^30,32,33,38,40,42,43^. Our estimated 5-year LC for WHO grade II (66.7%) and WHO grade III (53.1%) meningiomas is compatible with the majority of published data (Supplementary Table S2)^11,13,15,30,32,33,38,40,42,44–47^. Our 5-year and 10-year OS rates for each WHO grade, however, seem to be more favorable in comparison to published ones. This might be due to our low number of high-risk meningioma patients limiting statistical information. In addition, histological grading in older samples was not updated to the revised WHO grading system from 2016 influencing the indication for radiotherapy, the target volume, applied dose and probably the outcome^48^. Although concordance for histopathological grading of meningioma is relatively high, there is still some interobserver and interinstitutional discrepancy, which might lead to a bias in outcome^49^.

## Conclusion

In our cohort of mostly advanced or relapsed meningioma patients, radiotherapy showed an excellent prognosis with regard to OS and LC and acceptable HRQoL with low physician-reported toxicity. HRQoL deterioration should be considered against the risk of meningioma recurrence and may therefore guide decision making when opting for or against radiotherapy. Prospective studies should aim for improvement of HRQoL without worsening oncological outcome.

## Supplementary Information

Supplementary Information.

## Data Availability

The datasets generated during and/or analyzed during the current study are available from the corresponding author on reasonable request.
