# Supplementary material for: Health-related quality of life and clinical outcome after radiotherapy of patients with intracranial meningioma
Source: Sci Rep. 2022 Nov 17;12:19730. doi: 10.1038/s41598-022-24192-8 (PMC9672325; doi:10.1038/s41598-022-24192-8)
Supplement: Supplementary file 1 — Supplementary Information. [file 41598_2022_24192_MOESM1_ESM.pdf]

Health-related quality of life and clinical outcome after radiotherapy of patients with intracranial meningioma

Authors: Dominik Lisowski[1*], Jannik Trömel[2], Paul Lutyj[1], Victor Lewitzki[1], Philipp E. Hartrampf[3], Bülent Polat[1], Michael Flentje[1] and Jörg Tamihardja[1]

[1] Department of Radiation Oncology, University Hospital Wuerzburg, Wuerzburg, Germany

[2] Department of Internal Medicine, Leopoldina Hospital Schweinfurt, Schweinfurt, Germany

[3] Department of Nuclear Medicine, University Hospital Wuerzburg, Wuerzburg, Germany

* Correspondence: lisowski_d@ukw.de

Supplementary Figure S1

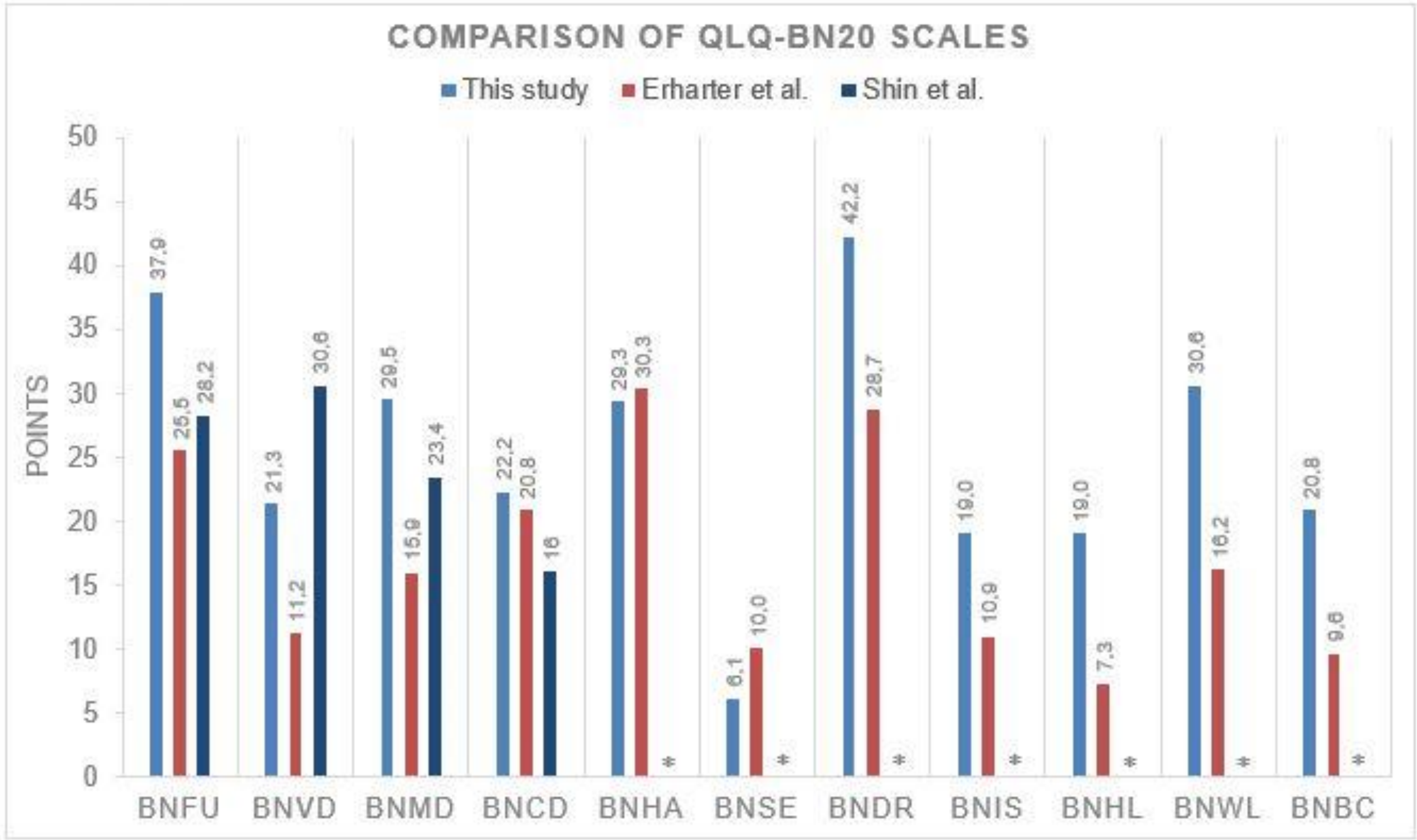

Comparison of QLQ-BN20 scales with previously published data. Higher scores suggests higher impairments and worse quality of life.
BNFU = Future uncertainty; BNVD = Visual disorder; BNMD = Motor dysfunction; BNCD = Communication deficit; BNHA = Headaches; BNSE = Seizures; BNDR = Drowsiness; BNIS = Itchy skin; BNHL = Hair loss; BNWL = Weakness of legs; BNBC = Bladder control.

*Data not published.

Supplementary Table S1. Baseline patient characteristics for survey (n = 81).

Variable	Responders (n = 49)	Non-responders (n = 32)	
Gender			p = 0.67
Male	16 (32.7%)	9 (28.1%)	
Female	33 (67.3%)	23 (71.9%)	
Age at time of radiation			p = 0.06
Mean (SD)	58.4 (9.4)	51.9 (17.5)	
Age at time of survey			p = 0.27
Mean (SD)	64.8 (9.3)	61.0 (17.5)	
Localization			p = 0.59
Skull base	31 (63.3%)	19 (59.4%)	
Cerebral falx	11 (22.4%)	10 (31.3%)	
Hemispherical convexity	6 (12.2%)	1 (3.1%)	
Optic nerve sheath	1 (2.0%)	2 (6.3%)	
Histology			p = 0.98
No specimen collected	17 (34.7%)	9 (28.1%)	
WHO Grade I	19 (38.8%)	16 (50.0%)	
WHO Grade II	8 (16.3%)	6 (18.8%)	
WHO Grade III	5 (10.2%)	1 (3.1%)	
Karnofsky Performance Status			p = 0.73
Median (range)	90 (50-100)	90 (40-100)	
KPS ≥90%	31 (63.3%)	19 (59.4%)	
KPS <90%	18 (36.7%)	13 (40.6%)	
Simpson Resection Grade (n = 56)	36 (73.5%)	20 (62.5%)	p = 0.88
Not known	5 (10.2%)	1 (3.1%)	
Grade I	1 (2.0%)	2 (6.3%)	
Grade II	5 (10.2%)	3 (9.4%)	
Grade III	2 (4.1%)	0 (0%)	
Grade IV	23 (46.9%)	11 (34.4%)	
Grade V	0 (0%)	3 (9.4%)	
Treatment			p = 0.62
Primary radiation	12 (24.5)	10 (31.3)	
Adjuvant radiation	9 (18.4)	5 (15.6)	
Relapse radiation	28 (57.1)	17 (53.1)	
Radiation modalities			p = 0.06
FSRT	21 (42.9%)	20 (62.6%)	
IMRT	25 (51.0%)	12 (37,5%)	
SRS	3 (6.1%)	0 (0%)	
WBRT	0 (0%)	0 (0%)	
Tumorsize			p = 0.63
Mean (SD)	2.7 (2.0)	2.9 (1.7)	
PTV			p = 0.37
Mean (SD)	78.7 (77.1)	99.4 (129.7)	
Total dose			p = 0.09
Median (IQR)	54.0 (54.0 - 60.0)	56.1 (54.0 - 60.7)	
Radiopeptide therapy			p = 0.04
Yes	6 (12.2%)	0 (0%)	
No	43 (87.8%)	32 (100%)	

FSRT = fractionated stereotactic radiotherapy; IMRT = intensity modulated radiotherapy; IQR = interquartile range; KPS = Karnofsky Performance Status; PTV = planning target volume; SD = standard deviation; SRS = stereotactic radiosurgery; WBRT = whole brain radiotherapy; WHO = World Health Organization.

Supplementary Table S2. Results of retrospective studies reporting LC and OS for meningioma patients undergoing radiotherapy.

Author	WHO grade	Number of patients	Radiation modality	Median single dose +/- range	Median total dose +/- range	5-year local control	10-year local control	5-year overall survival	10-year overall survival
This study	I/NA<br>II<br>III	38/43<br>20<br>18	Photon	1.8 Gy (1.7–4.0 Gy)$^{\times}$<br>1.8 Gy (1.8–2.1 Gy)$^{\times}$<br>1.8 Gy (1.8–2.15 Gy)$^{\times}$	54.0 Gy (20–66 Gy)$^{\times}$<br>60.0 Gy (54–66 Gy)$^{\times}$<br>60.0 Gy (54–65 Gy)$^{\times}$	92,2%<br>66,7%<br>53,1%	86,6%<br>46,7%<br>42,5%	88,8%<br>100,0%<br>81,9%	80,7%<br>87,1%<br>44,1%
Soldà et al.	I/NA	128/94	Photon	30–33 fractions	50.0–55.0 Gy	93,0%	86,0%	93,0%	84,0%
Hamm et al.$^{\sim}$	NA<br>I<br>II<br>III	95<br>113<br>10<br>6	Photon	SRT: 1.8–2.0 Gy; SRS: single fraction	SRT: 55.8 Gy (50.4–67.5 Gy); SRS: 12.8–18 Gy prescribed to the 80% isodose	ND	ND	92,9%	ND
Combs et al.$^{\sim}$	I<br>II/III	234<br>20/15	Photon	1.8 Gy (1.6–5 Gy)	57.6 Gy (25.0–68.0 Gy)	95,0%<br>81,0%	91,0%<br>53,0%	95,0%	90,0%
Tanzler et al.	I	146	Photon	1.8 Gy	52.7 Gy (45.0–55.8 Gy)	97,0%	96,0%	87,0%	79,0%
Wegner et al.	I<br>II	46<br>10	Photon	5 fractions (1–5)	25 Gy (13–27.5 Gy) prescribed to the 80%-100% isodose	ND	88,0%	ND	86,0%
Milker-Zabel et al.	I/NA<br>II	153/138<br>26	Photon	1.8 Gy	57.6 Gy (45.0–68.0 Gy)	90,5%<br>89,0%	89,0%<br>67,0%	94,7%	90,0%
Debus et al.	I/NA	121/59	Photon	1.8 Gy	56.8 Gy* (± 4.4 Gy)	ND	ND	97,0%	96,0%
Pasquier et al.	II<br>III	82<br>37	Photon	2.0 Gy (1.8–2.0)	54.6 ± 5.1 Gy* (40.0–66.0 Gy)	62,0%<br>48,0%	ND	67,5%<br>60,0%	ND
Hug et al.	II<br>III	15<br>16	Photon/Photon+Carbon Ion	1.8–2.0 Gy/CGE	62 Gy/CGE* $^{\#}$ (50–68 Gy/CGE)<br>58 Gy/CGE* $^{\#}$ (40–72 Gy/CGE)	38,0%<br>52,0%	19.0%†<br>17.0%†	89,0%<br>51,0%	89.0%†<br>51.0%†
Adeberg et al.	II<br>III	62<br>23	Photon/Photon+Carbon Ion	1.8–3.0 Gy$^{\#}$	57.6 Gy (30.0-68.4 Gy)$^{\#}$	50,0%<br>13,0%	ND	81,0%<br>53,0%	ND
Boskos et al.	II/III	19/5	Photon/Photon+Carbon Ion	1.8–2.0 Gy/CGE$^{\#}$	68 Gy/CGE$^{\#}$	46,7%	46.7%†	53,2%	42.6%†
Stafford et al.	I<br>II<br>III	168<br>13<br>9	Photon/Gamma-Knife	ND	16 Gy (12–36 Gy) prescribed to the 33-90% of isodose	93,0%<br>68,0%<br>0,0%	ND	92,0%<br>76,0%<br>0,0%	ND

CGE = cobalt Gray-equivalent; LC = local control; NA = no histology available; ND = no data available; OS = overall survival; SRS = stereotactic radiosurgery; SRT = stereotactic radiotherapy; × median dose for FSRT and IMRT only; * mean dose; † results after 8 years; # combined dose of photon and carbon ion radiation; ~ only skull base meningioma.
